# Classification of rotation-invariant biomedical images using equivariant neural networks

**DOI:** 10.1038/s41598-024-65597-x

**Published:** 2024-07-01

**Authors:** Karl Bengtsson Bernander, Ida-Maria Sintorn, Robin Strand, Ingela Nyström

**Affiliations:** 1 Centre for Image Analysis, Department of Information Technology, Uppsala University, Uppsala, Sweden; 2 Vironova AB, Stockholm, Sweden

**Keywords:** Applied mathematics, Virology, Transmission electron microscopy

## Abstract

Transmission electron microscopy (TEM) is an imaging technique used to visualize and analyze nano-sized structures and objects such as virus particles. Light microscopy can be used to diagnose diseases or characterize e.g. blood cells. Since samples under microscopes exhibit certain symmetries, such as global rotation invariance, equivariant neural networks are presumed to be useful. In this study, a baseline convolutional neural network is constructed in the form of the commonly used VGG16 classifier. Thereafter, it is modified to be equivariant to the p4 symmetry group of rotations of multiples of 90° using group convolutions. This yields a number of benefits on a TEM virus dataset, including higher top validation set accuracy by on average 7.6% and faster convergence during training by on average 23.1% of that of the baseline. Similarly, when training and testing on images of blood cells, the convergence time for the equivariant neural network is 7.9% of that of the baseline. From this it is concluded that augmentation strategies for rotation can be skipped. Furthermore, when modelling the accuracy versus amount of TEM virus training data with a power law, the equivariant network has a slope of − 0.43 compared to − 0.26 of the baseline. Thus the equivariant network learns faster than the baseline when more training data is added. This study extends previous research on equivariant neural networks applied to images which exhibit symmetries to isometric transformations.

## Introduction

### Classification of biomedical images

**Figure 1:**
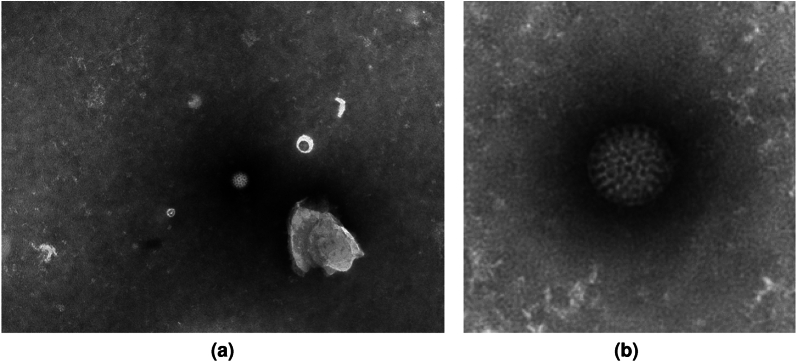
Raw image of a rotavirus from the TEM virus dataset. (**a**) The virus particle is located in the center of the image. (**b**) Cutout image of the rotavirus particle.

Transmission Electron Microscopy (TEM) can be used to visualize pathogens and subcellular structures in order to identify the infectious agents in the samples^1^. TEM is commonly used to characterize new pathogens when combined with other methods. As an example, starting in the year 2020, the COVID-19 pandemic impacted the world, resulting in millions of deaths. TEM played an important role in identifying the cause as the SARS-CoV-2 virus, a type of corona virus, with its characteristic corona of glycoproteins^2^. With a sufficient amount of TEM images of viruses annotated by experts, it is possible to deploy machine learning methods for automatic classification of new samples. However, it can be difficult to find the experts and allocate the data needed^3^. Thus, methods that can reduce the amount of data needed are useful in such scenarios. Since new epidemics with new or unknown pathogens are expected in the future, methods for faster and more accurate characterization are in great demand. In this article, a dataset of negative stain TEM images containing different types of viruses of which several exhibit rotational symmetries is used exemplified in Fig. 1. An example of a related biomedical image analysis task is the classification of different types of blood cells from light microscopy images. Here, similar symmetries manifest in the images. Furthermore, large amounts of expert annotated data is needed for automatic and faster classification to make it more accessible.

### Equivariant neural networks

In the last decade, convolutional neural networks (CNNs) have been very successful in computer vision problems, such as classification and segmentation. Although vision transformers are becoming more and more competitive^4^, CNNs are likely to continue to be utilized, in part due to their familiarity in the biomedical image analysis community.

One convenient property of CNNs is their translation equivariance. This means that if the input to a convolution layer is translated, then the output is accordingly shifted up, down, left or to the right. Equivariance is commonly expressed in the following way:

1 $$\begin{aligned} T(C(x)) = C(T(x)), \end{aligned}$$

where *x* is the input to a convolution layer *C*, and *T* is a transformation, such as a translation, of the input data or feature map. Here, the convolution operator commutes with the transformation. It is also possible to construct invariant classifiers, meaning that the output of the network is identical no matter the translations of the signals in the input image.

However, this property does not hold for other isometric, i.e., distance-preserving, transformations such as rotations or reflections. Therefore, substantial research efforts have been expended to design such architectures. One of the most common approaches is group-equivariant neural networks^5^, which extends the convolution operator to a specific symmetry group. This operation is called G-convolution.

G-convolutions generalize the convolution operator to not only perform translations of the kernel *k* across the input, but isometric, i.e. distance-preserving, transformations as well. For the input layer and the transformation *g*, the G-convolution is defined as follows:

2 $$\begin{aligned} z(x,y) = k * f(x,y)[g] = \sum _{i=-a}^{a}\sum _{j=-b}^{b}k(i,j)f[g^{-1}(x-i,y-j)], \end{aligned}$$

where *x* and *y* are image coordinates, *i* and *j* are kernel coordinates in the window of size $[-a,a]$ by $[-b,b]$, and *z* is the output. The output is a stack of feature maps for transformations *g* in the symmetry group *G*.

As an example, the symmetry group can be the p4 group, i.e., discrete rotations of 0, 90, 180, and 270°. For the p4 group, the group convolution layer will apply rotated versions of the convolution kernels to the input and save the results in a feature stack. Since the weights are thereby shared over the stack, there is a reduction of the number of weights in the networks. This is in contrast to CNNs which need to learn each rotation separately, which in itself constitutes an increased risk of overfitting to the data.

There are other ways of achieving rotation equivariance, including the method of steerable filters^6^, which uses basis filters to achieve arbitrary angular resolutions. Another approach is CFNET, which uses conic convolutions and discrete Fourier transforms^7^. It is also possible to rotate the feature maps themselves^8^. The general E(2)-equivariant steerable CNNs unifies G-convolutions, steerable filters, and other methods in a framework on the ${\mathbb {R}}^2$ Euclidean space^9^. In fact, the E(N)-Equivariant Steerable CNNs framework accomplishes similar qualities on the ${\mathbb {R}}^3$ Euclidean space^10^.

### Empirical results

Recent research into equivariant neural networks has demonstrated that they are generally more advantageous in comparison with CNNs. This holds when the data they are trained on is symmetrical to, e.g., rotations and reflections. Biomedical datasets are especially useful in these cases, because classification is invariant to the global orientation of the samples under a microscope. Similar symmetries manifested on more local scales, such as the shapes and distributions of organelles or virus envelopes, could also aid classification. A rotation-equivariant VGG16 architecture has shown to improve accuracy on the test set for cytological diagnosis of oral cancer, resulting in less overfitting^11^. It was also shown that common data augmentation strategies could be skipped. Similar results were seen for mitosis detection, vessel segmentation, and cell boundary segmentation^12^. The images in these datasets were taken using transmission light microscopy, reflected light microscopy and transmission electron microscopy, respectively. Similarly in 3D for MNIST data projected onto the sphere, equivariant models perform equally well or better than non-equivariant models combined with data augmentation^13^.

Another improvement of equivariant models over baseline CNNs is the convergence speed when training. In the setting of semantic instance segmentation, this effect was demonstrated for a rotation-equivariant version of U-net with a discriminative loss function^14^. A similar effect has been seen in reinforcement learning, when the network is constrained to be equivariant to certain symmetries in the joint action-state space of a Markov decision process^15^. While not a strictly equivariant model, initializing CNNs with rotation-invariant Zernike moments improves the convergence speed in facial classification^16^.

Furthermore, equivariant models are more efficient in learning from fewer training examples for mitosis detection, nuclei segmentation, and tumor classification^17^. This is consistent with a trend seen in learning molecular dynamics^18^. Here, a log-log plot of the error versus the amount of data reveals a steeper slope for the equivariant neural network in comparison to the baseline. This effect is not seen when comparing other neural networks architectures^19^.

### Data efficiency

The area of more efficient learning from data is given extra attention. As the amount of data and the number of network parameters increase over the years, so does the time spent on training and developing the models. While distributing the computations over, e.g., Graphical Processing Units (GPUs) does much to alleviate the issue, models still typically take weeks or months to optimize, even when many GPUs are accessible. The search for optimal hyperparameters and network architectures necessitates a large number of experiments, which is often too costly to do with larger models or with the full amount of data. The characterization of the model behaviour over different ranges of training data or model sizes is called predictable scaling^19^, which is used in e.g. the development of large scale language models^20,21^ or computer vision problems^22^. The procedure involves doing the architecture and hyperparameter searches in smaller data or architecture regimes, determining power laws to get a sense of the full model’s performance, and finally training the full model. This same procedure could be very useful for large-scale problems in biomedical settings. This study aims to show that it would be even more efficient using equivariant neural networks.

### Aims

Previous studies indicate that equivariant networks offer benefits over CNNs, including more efficient learning from new training data, faster training, skipping data augmentation steps and higher accuracy on test sets. However, it is currently not clear how general these qualities are. More research is needed using more network architectures and biomedical image datasets, and the intention behind this study is to fill this knowledge gap with more empirical results and analysis.

## Methods

### Approach

**Figure 2:**
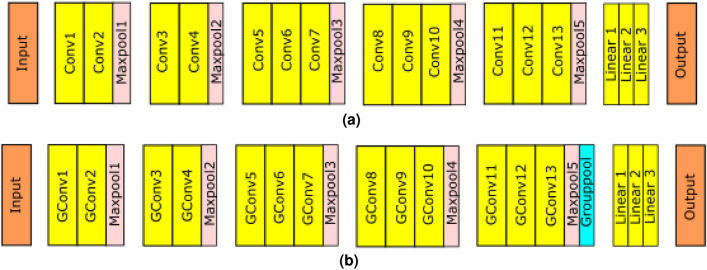
The VGG16 architectures used in the experiment. Subfigure (**a**) shows the baseline, and (**b**) shows the group-equivariant version.

To contrast and compare the equivariant version of a CNN with a baseline version, the high-performing and well established VGG16 architecture^23^ was selected. First, a baseline network using ordinary convolutions was constructed. The details of the architecture and hyperparameters can be seen in Table 1. Thereafter, an additional network was implemented by replacing the convolutions with group convolutions in each layer of the network. An additional group pooling layer to make the classifier invariant was inserted before the linear layers. The new network, called GCNN, was designed to be equivariant to the transformations of the p4 group consisting of multiples of 90° rotations. This symmetry group was chosen to match commonly used transformations when performing data augmentation. Both data augmentation by rotations and rotation-equivariant networks exploit global invariance to rotations, as the orientation of the sample under a microscope is without meaning. Also, this group avoids the interpolation artifacts associated with finer angular resolutions. This was achieved by using the E2CNN library on top of Pytorch^9^. Since the size of the feature maps were increased by four for the p4 group, the number of channels in the GCNN was halved to keep the number of weights the same as for the baseline architecture. Both the baseline and the group-equivariant models are illustrated in Fig. 2.

To measure convergence time, the following metric was used. First, the highest accuracy on the validation set is saved. Then, the first epoch which reaches at least 95% of the highest accuracy is saved. The accuracy at this epoch is called the stable accuracy. Finally, the time when finishing this epoch from the initial time is saved. This is called the time until stability. The experiments were deployed on the Swedish NAISS cloud computing system, using an A40 GPU and 64 GB system memory. To aid reproducibility efforts, the code used in the experiments is provided here: https://github.com/kbbernander/TEM-equivariance.

**Table 1:** Settings for the CNN architecture and training procedure.

Parameter	Setting
Loss function	Cross entropy
Weight initialization	He
Optimizer	Adam
Learning rate	0.00001
No. of epochs	100
Activation functions	ReLu
Dropout	0.5 between linear layers
No. of channels	32-32-64-64-128-128-128-
	256-256-256-256-256-256
Convolution layers	Layers 1–12: (3,1,1)
(Size, stride, padding)	Layer 13: (4,0,0)
Maxpooling layers	Layers 1–4: (2,2,0)
(Size, stride, padding)	Layer 5: (13,1,0)
Linear layer parameters	(256,4096)–
(Input, output sizes)	(4096,4096)–(4096,14)

### Datasets

The first dataset consists of images of 14 different species of viruses imaged by transmission electron microscopy (TEM). The dataset was prepared for machine learning test purposes of virus classification and published in^24,25^, where the image set is described in detail. In brief, samples were prepared by treating them with 10% phosphate-buffered saline, before added to carbon-coated TEM grids and stained with 2% phosphotungstic acid (PTA) following standard negative stain sample preparation procedures. Images were acquired at different magnifications giving pixel sizes ranging from 0.26 to 5.57 nm, with either a LEO (Zeiss, Oberkochen, Germany) microscope mounted with a Morada (Olympus) camera or a Tecnai 10 (FEI, Hillsboro, OR, USA) with a MegaView III (Olympus, Münster, Germany) camera. All images were resized using a Lanczos-3 kernel to have a common pixel size of 1 nm. Each sample is labelled with its corresponding virus type. In the experiments, cropped images of size $256 \times 256$ pixels centered around the virus particles were used. Figure 1 presents an example of a virus image with a rotavirus particle, where global rotation invariance is exhibited in both the raw image and cutout.

The training data was re-balanced so that each class has 93 particles, which is the lowest amount found in any original class. For classes exceeding this number, augmented images were first removed and then images were randomly removed. This is called the modified dataset. An augmented version of the modified training set was also created. Each image in the training set was rotated by 90, 180, and 270°, as well as reflected over one axis. This resulted in eight images in total for each original image. The validation and test sets were left unchanged. The training, validation and test sets were established at the image level to prevent data leakage.

To collect further results on data with similar characteristics as the TEM data, i.e. the rotation invariance of orientations under a microscope, the BloodMNIST dataset from the MedMNIST collection^26^ was used as well. The samples were collected and labelled at the Core Laboratory at the Hospital Clinic of Barcelona^27^. To acquire the light microscopy images, the analyzer CellaVision DM96 was used. The images are of size 28 by 28 pixels of healthy human peripheral blood cells divided into eight different classes. The training set consists of 11,959 images, the validation set of 1711 images, and the test set of 3420 images. The RGB images were converted to greyscale. An augmented training set was prepared with the same transformations as for the TEM data.

### Main experiments

Since one of the aims was to determine if a rotation-equivariant network would outperform a baseline network in terms of, e.g., convergence speed and classification accuracy, the TEM virus modified training data was used. Three comparisons were created based on four tests. In the first comparison, the baseline network with data augmentation consisting of p4 rotations as well as reflections in one axis is expected to outperform the baseline without data augmentation. This is referred to as Control 1. Secondly, the GCNN without data augmentation is expected to outperform the baseline network with data augmentation, because the GCNN using the p4 symmetry group will match the rotations of the augmented data. In contrast, the CNN has to devote parameters to learn each separate rotation in the augmented data, increasing the risk of overfitting. This is the main hypothesis. Finally, the GCNN is not expected to benefit much when adding data augmentations of the same type as in the symmetry group, although reflections are also present in the data augmentation. This is referred to as Control 2. Five separate runs were made for each experiment, and the means of the runs were calculated. To further validate the results, additional experiments following the same procedure as described above were carried out on the BloodMNIST dataset, using the p4m group which includes reflections about one axis in addition to the transformations of the p4 group.

### Varied amounts of training data

Experiments were also designed to investigate how the availability of training data affects the accuracy on the validation set. The TEM virus training data was varied in steps of around 200 samples in the interval between 126 and up to the full amount, 1302 samples. The experiments were performed for both the baseline network and the GCNN on the unaugmented training set.

### Cross-validation

Finally, cross-validation was performed to control for overfitting or selection bias on the TEM virus data. The folds were constructed by merging the training and test sets and then dividing the merged dataset into five folds with data selected randomly. The training folds consisted of 100 images per class while the test folds held 23 images per class. Identical to the main experiments, each image in the training set was rotated by 90, 180, and 270°, as well as reflected over one axis.

## Results

**Table 2:**
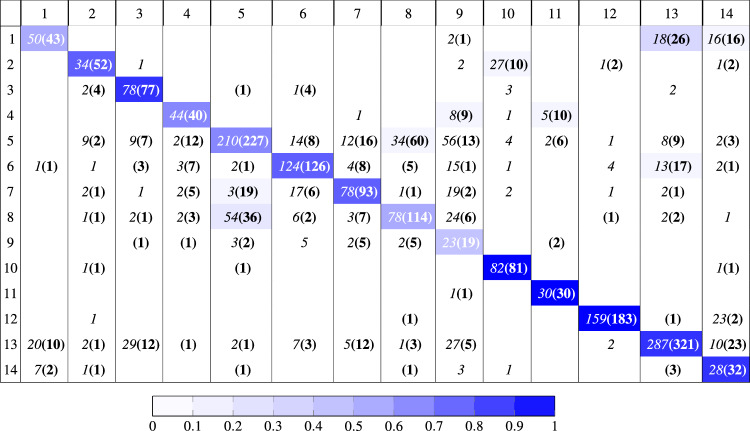
Confusion matrix when data augmentation is used on the TEM virus data. The numbers indicate the number of classified examples. Results from using the CNN are shown without parentheses, while results from using the GCNN are shown inside parentheses and in bold. Results indicating zero classifications are omitted. The colors indicate the percentage classified of the total class examples for the GCNN. The rows indicate the actual class, while the columns indicate the predicted class.

### Main experiments

**Figure 3:**
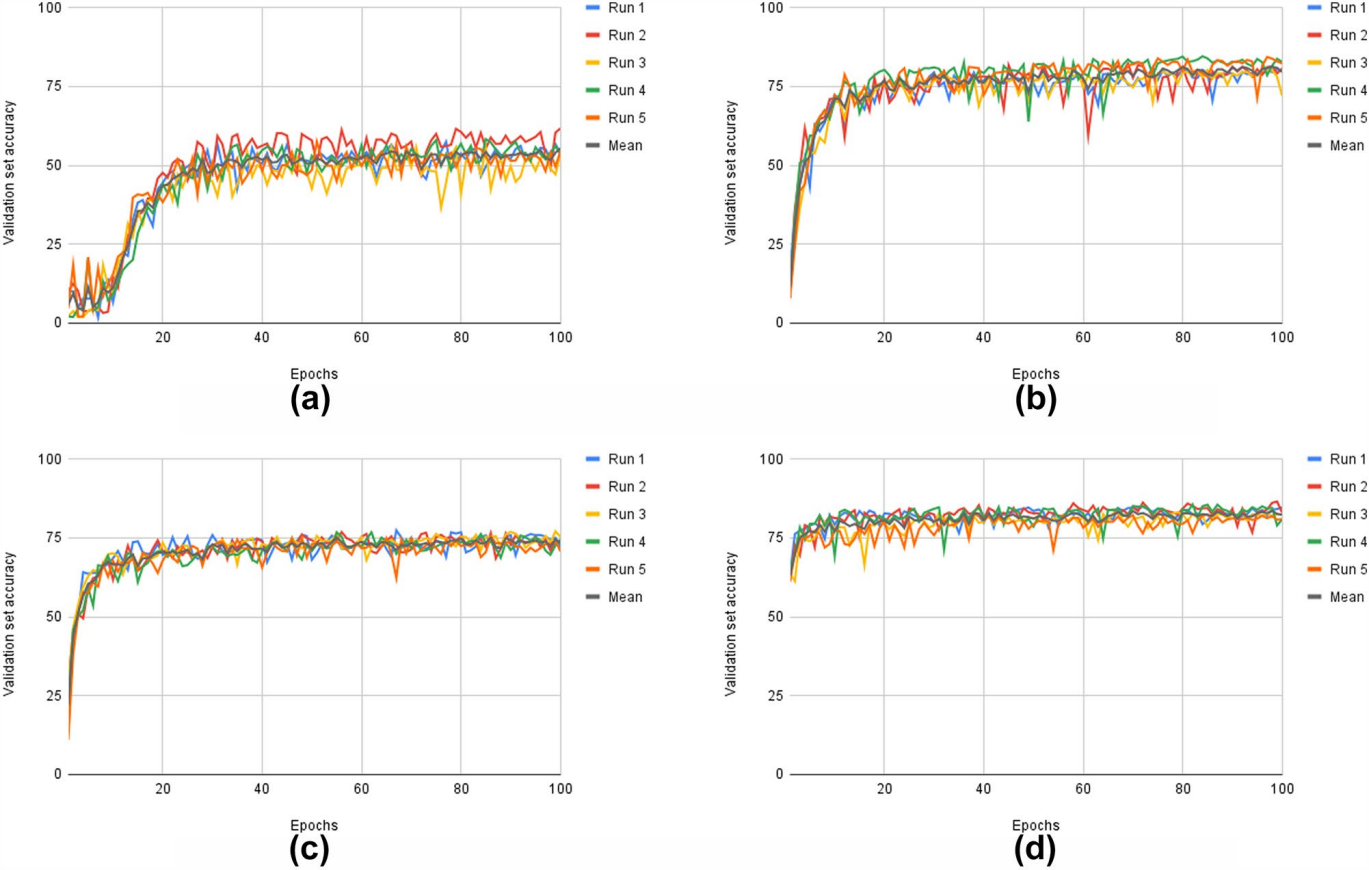
Results on the TEM virus data for five different runs and their mean values. (**a**) Results for the CNN on the unaugmented dataset. (**b**) Results for the GCNN on the unaugmented dataset. (**c**) Results for the CNN on the augmented dataset. d) Results for the GCNN on the augmented dataset.

The results on the modified unaugmented and augmented training sets are shown in Fig. 3. The results are summarized in Table 3. It is evident that the GCNN without data augmentation outperformed the baseline network with data augmentation (82.43% top accuracy on the validation set compared to 76.59%, an increase of 7.6%). These results are statistically significant with $p = 0.0081$ in a paired t-test. The confusion matrix for the first experimental run for both the augmented CNN and the unaugmented GCNN on the test set are seen in Table 2. It can be seen that the GCNN is slightly more accurate. For instance, the GCNN has higher number of true positives for 8 of the 14 classes. From the values in the confusion matrices, the average sensitivity can be calculated as 0.67 and 0.71, respectively. Similarly, the average specificity is 0.97 and 0.98, respectively.

Stable accuracy was reached in epoch 25 after 2440 seconds for the GCNN, while it took 21 epochs and 10581 seconds for the baseline network with data augmentation. Thus, the GCNN converged in 23.1% of the corresponding time of the baseline network. The numbers are statistically significant with $p = 0.0021$ in a paired t-test.

Furthermore, there was a clear benefit in terms of validation set accuracy when adding data augmentation to the baseline network (76.59% compared to 57.99%). This confirms the hypothesis of the Control 1 comparison. However, this resulted in a slower convergence speed. Stable accuracy was reached in epoch 21 after 10581 seconds for the CNN with augmented data, while it took 36 epochs after 2076 seconds without data augmentation.

Finally, there was a benefit in terms of validation set accuracy in adding data augmentation to the GCNN (84.49% compared to 82.43%). This is not in line with the hypothesis of the Control 2 comparison. Stable accuracy was reached in epoch 7 after 3894 seconds for the GCNN with augmented data, while it took 25 epochs after 2440 seconds without data augmentation.

The results on the BloodMNIST dataset are shown in Table 4. It can be seen that there was no gain in accuracy on the validation set when comparing the GCNN on the unaugmented training data with the CNN on the augmented training data. Both conditions resulted in accuracies of around 86–88%. Still, there was significant speedup for the GCNN. It converged to the stable accuracy in 4008 seconds, which is 7.9% of the 50,821 s it took for the CNN.

**Table 3:** Results from the main experiments on the TEM virus dataset on the validation set.

Augmentation scheme	CNN	GCNN
No augmentation		
Best accuracy	57.99 ± 2.27	82.43 ± 1.95
Epochs to stability	36	25
Time to stability	2076 ± 336	2440 ± 718
Data augmentation		
Best accuracy	76.59 ± 0.76	84.49 ± 1.63
Epochs to stability	21	7
Time to stability	10,581 ± 2672	3894 ± 717

The numbers are averages and standard deviations over five runs. The time to stability is in seconds.

**Table 4:** Results from the main experiments on the BloodMNIST dataset on the validation set.

Augmentation scheme	CNN	GCNN
No augmentation		
Best accuracy	88.20 ± 0.43	87.26 ± 0.59
Epochs to stability	36	10
Time to stability	8877 ± 1313	4008 ± 805
Data augmentation		
Best accuracy	86.73 ± 0.32	87.89 ± 0.60
Epochs to stability	26	14
Time to stability	50,821 ± 4936	43,663 ± 11020

The numbers are averages and standard deviations over four runs. The time to stability is in seconds.

### Varied amounts of training data

**Figure 4:**
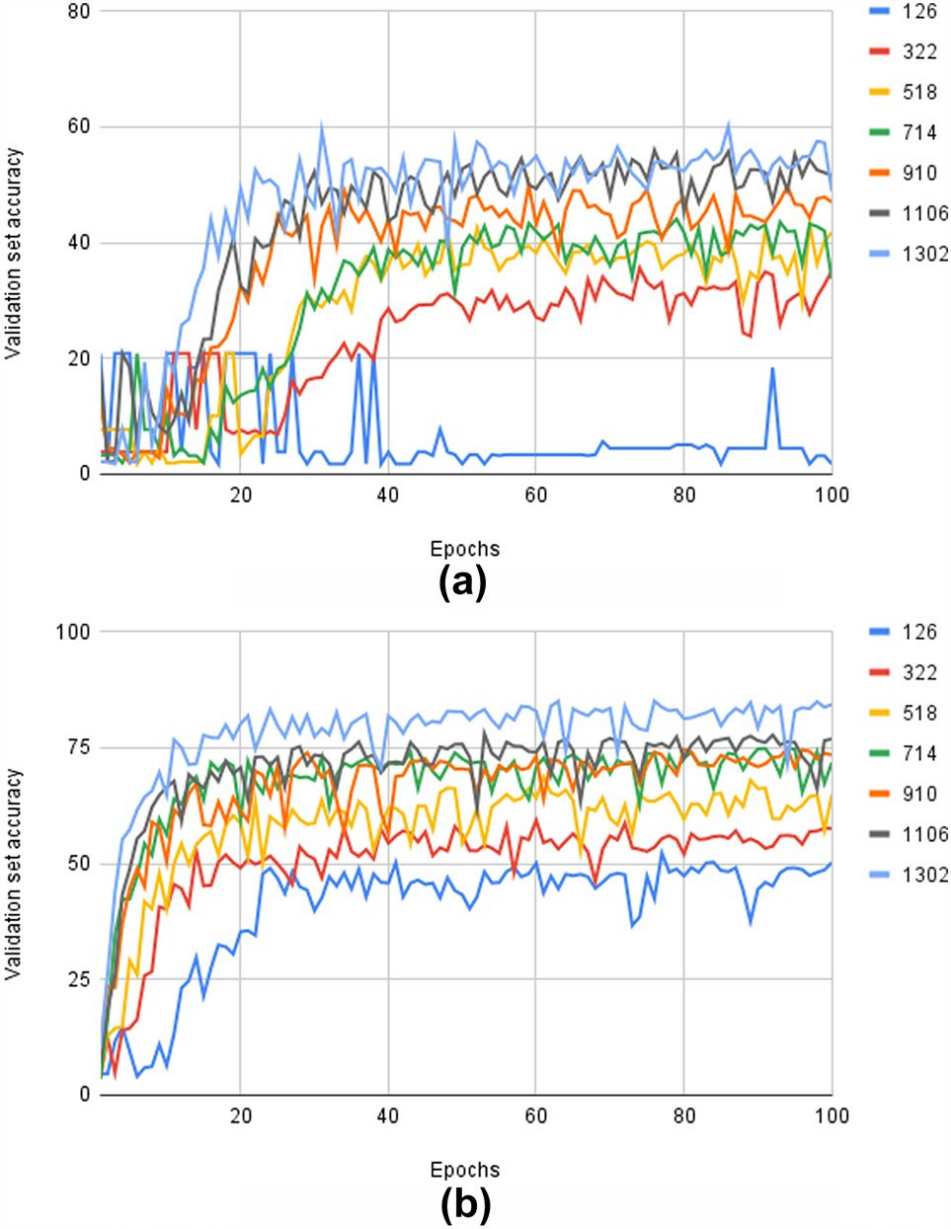
Validation set results on the modified unaugmented TEM virus dataset for varying amounts of training data. (**a**) Results for the CNN. (**b**) Results for the GCNN.

The results from the experiments on the TEM virus data when varying the amount of unaugmented training data are shown in Fig. 4. It can be seen that more training data yields higher accuracy on the validation set for both networks. Also, the GCNN consistently reaches higher accuracy in contrast to the CNN. As an example, when the number of training samples is 714, the top accuracy for the CNN is 44.06%, while the corresponding accuracy for the GCNN is 74.88%.

Thus, the GCNN is more data efficient than the baseline network, which can be shown quantitatively. For any power law

3 $$\begin{aligned} y = ax^k, \end{aligned}$$

taking its logarithm and rearranging leads to the following:

4 $$\begin{aligned} log (y) = log (a) + k*log(x). \end{aligned}$$

**Figure 5:**
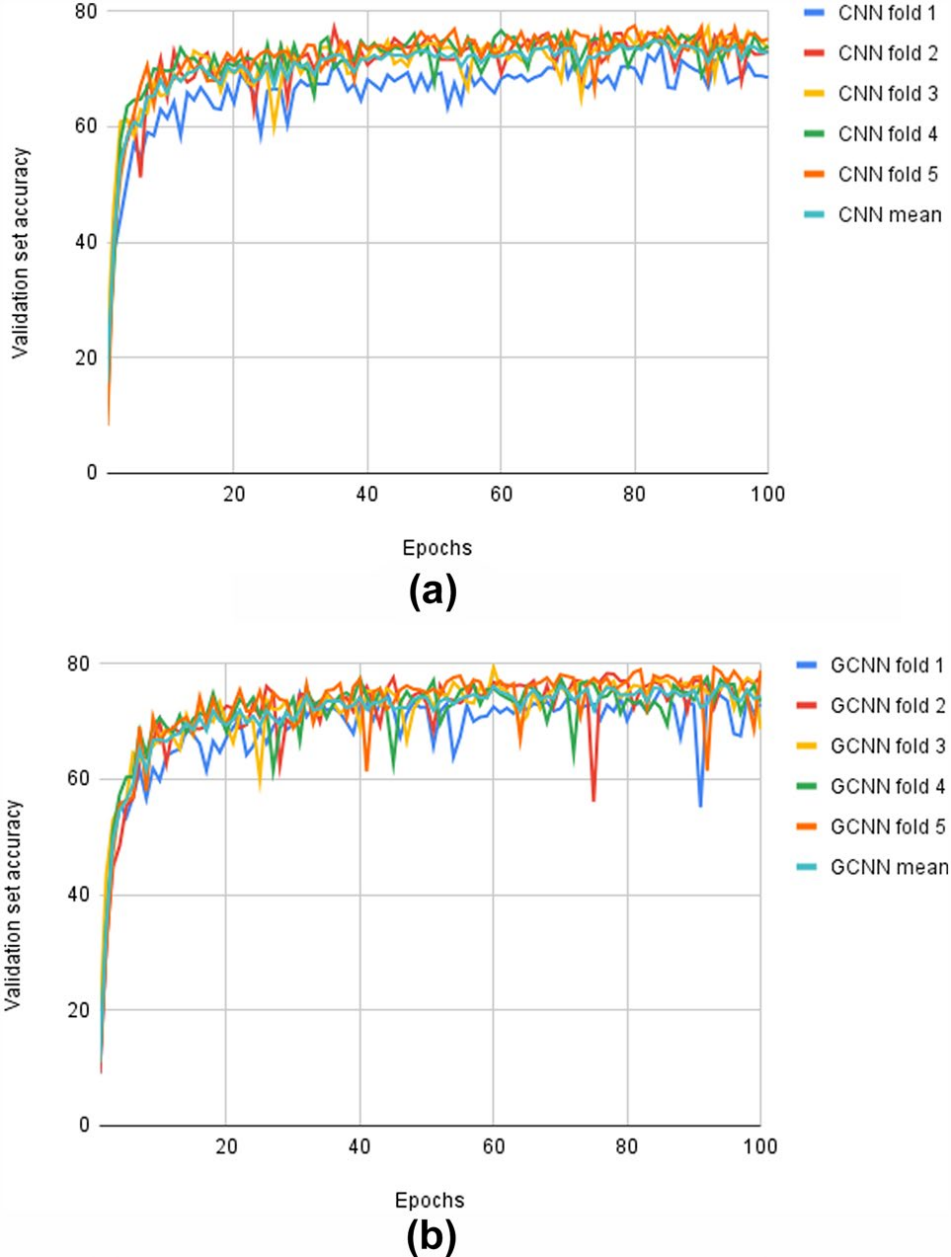
Cross-validation results on the TEM virus dataset on the validation set. (**a**) Results for the CNN. (**b**) Results for the GCNN.

**Figure 6:**
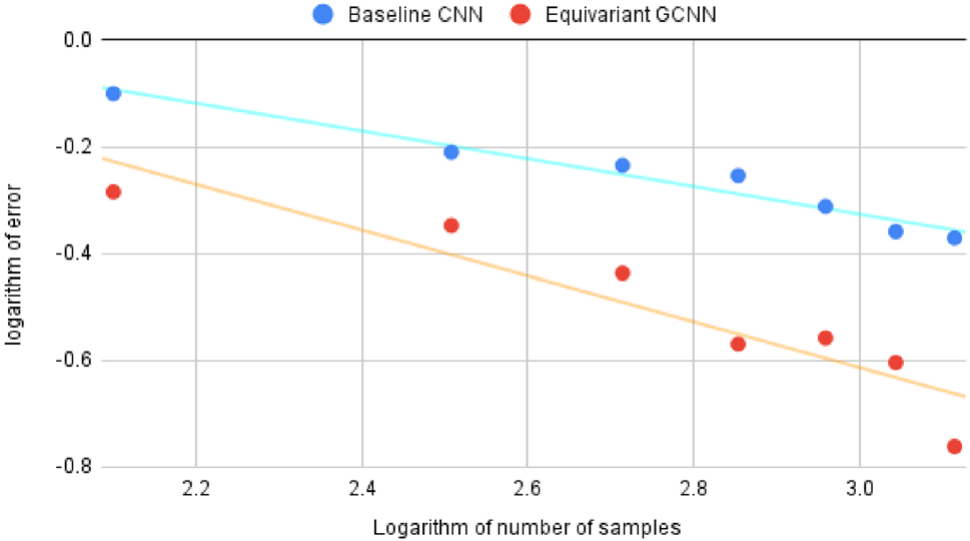
The error on the TEM virus test set for varying amounts of training data. The CNN has a slope of − 0.26 in the log-log scale, while the GCNN has a slope of − 0.43.

The parameters of this model can be found using linear regression. This procedure has been performed in Figs. 5, 6, which shows a log-log plot of the end errors on the test set versus the amount of training samples. It can be seen that the baseline network has $k = -0.26$, while the GCNN has $k = -0.43$. Thus, the GCNN reduces the error on the test set faster than the baseline when more training data is added.

### Cross-validation

The results of the cross-validation experiments for the TEM virus data are shown in Fig. 5. The mean accuracy on the test set is 87.39% for the GCNN. For the CNN, this number is 85.03%. It can also be seen that there is a consistently higher accuracy for the GCNN without data augmentation as compared to the CNN with data augmentation.

The standard deviation is 3.70 for the CNN and 4.18 for GCNN. Thus there is a significant variance in terms of test set accuracy depending on which folds are uses as training data. This could indicate that the dataset is highly varied in terms of, for example, illumination or the classes and number of particles present. Hence, the training process may be sensitive to what images are present in the training data.

In addition, the accuracies are generally higher when compared to the original data. A possible explanation is that a form of data leakage has occurred when the original training and test data have been mixed, because the sets in the original setup were kept independent at the image level.

## Discussion

The TEM virus data exhibits rotation invariance on a global scale. Hence, we assume that rotation-equivariant neural networks are suitable for faster and more data-efficient learning for such data, which the experiments support. In fact, the GCNNs generally converge by on average 23.1% of that of the CNN, and yield higher top accuracy on the validation set by on average 7.6%. Adding data augmentation to GCNNs increases the accuracy by a further 2.5%. This could be explained by the additional augmentations consisting of reflections in one axis, which could be learnt by the GCNN. The GCNN is designed to be equivariant to the p4 group consisting of only rotations and translations, not reflections. Also, even if the number of epochs are fewer, the amount of data is larger, which means more time is required until stability by a factor of around 1.6. If the goal is the highest accuracy possible, then data augmentation should be kept. On the other hand, if the goal is faster training, then the data augmentation steps can be skipped. On the BloodMNIST dataset for the same conditions as with the TEM virus data there was no boost in validation set accuracy when comparing the GCNN to the CNN, but the time until stability was much lower, about 7.9% of that of the CNN.

 Furthermore, for the TEM virus data, the GCNNs learn faster when increasing the amount of training data in contrast to the CNNs. This is demonstrated in the modelling of a power law through log-log plots where the GCNN and the CNN have slopes of − 0.43 and − 0.26, respectively.

The results were different on the datasets in terms of validation set accuracy. There was no improvement on BloodMNIST, while an improvement of 4.7% on the TEM virus dataset. The improvements in time until convergence were however consistent for both cases. This confirms previous research, which indicate that equivariant networks often improve over baseline CNNs, but with unknown quality of the improvement, e.g., higher accuracy, improved data efficiency or faster convergence speed.

Since equivariant models scale differently than CNNs on symmetrical data, this creates the possibility for general methods of development of high-performance classifiers. This knowledge means models could be trained in smaller data regimes to characterize a power law. The law would then predict the loss on the final optimized model. Searches for optimal network architecture and hyperparameters could be made on models using a small fraction of the training data. The final model could finally be trained using all the training data available. This would enable more model optimizations, save time in the development process and lower electricity consumption and emissions, to an even higher degree than if the same process were applied to a CNN architecture. The datasets should exhibit symmetries such as rotations and have large amounts of training examples. The equivariant network should incorporate these symmetries by design. This could be used in, for example, classification of individual cells, such as diagnosis of oral cancer in brush cytology. Other examples could include tissue analysis, such as diagnosis of cancer in breast tissue from biopsies. This could be advantageous since the orientation of tissue under microscopes is without meaning, similarly as for cytological analysis.

In addition to supporting the characterization of equivariant neural networks with new empirical results, our experiments advance the specific domain of more efficient analysis of TEM data for virus identification. This could be useful when a new pandemic arises as such a situation typically means a lack of data combined with a high need for visualization and characterization of the new pathogens, in combination with other methods. For diagnosis of pathogens in clinical practice, more sensitive molecular methods would be needed.

Future research will interpret the effects seen in these experiments. This includes further optimizations such as batch normalization and regularization. More network architectures and hyperparameters should be selected. The capabilities of visual transformers for biomedical image analysis should be investigated as well, and class activation maps to visualize the relevant feature of the data should be added. Scaling behavior under other conditions, e.g. when using different datasets, is also highly interesting. A natural extension of this study is to vary the symmetry groups used in the equivariant networks, such as the p4m group which is equivariant to reflections in one axis as well as multiples of 90° rotations, even though it is more costly in terms of computations. This work has recently been initiated^28^.

## Data Availability

The TEM virus data used in the current study are publically available in the Mendeley Data repository^25^. Similarly, the BloodMNIST data is found in the public Zenodo data repository^29^.
